# In Search of Suitable Breeding Sites: Habitat Heterogeneity and Environmental Filters Determine Anuran Diversity of Western Madagascar

**DOI:** 10.3390/ani13233744

**Published:** 2023-12-04

**Authors:** Nanäa Mausberg, Kathrin H. Dausmann, Julian Glos

**Affiliations:** Institute of Cell and Systems Biology, Universität Hamburg, Martin-Luther-King Platz 3, 20146 Hamburg, Germany; kathrin.dausmann@uni-hamburg.de (K.H.D.); julian.glos@uni-hamburg.de (J.G.)

**Keywords:** community ecology, amphibians, oviposition, pond ecology, species assemblages

## Abstract

**Simple Summary:**

Amphibian biodiversity is declining around the globe and habitat destruction and fragmentation impact two-thirds of all amphibians. The distinct characteristics of amphibians, such as low mobility and permeable skin, make them more susceptible to environmental influences than other taxa. We have shown that within the small area of the Kirindy Forest in Madagascar, environmental differences influenced the selection of breeding sites by frogs to such an extent that the composition of the anuran larvae assemblage differed vastly between individual ponds. The diversity of breeding sites increases species richness by serving the needs of multiple species. Characteristics such as pond size, vegetation, and time until desiccation of ponds should be considered when designing conservation action plans to protect a wide variety of species. If breeding sites become unavailable because of a shift in environmental gradients, not only amphibian diversity but the whole ecosystem could be harmed.

**Abstract:**

Environmental filtering shapes animal communities by preventing the colonization and persistence of certain species in a given habitat. More heterogenous environments are presumed to support a greater number of species and, consequently, increased species diversity, as environmental filters are also likely more heterogenous. Amphibians are especially sensitive to environmental influences due to distinct characteristics like permeable skin and low mobility. By analyzing the species richness and assemblage composition of tadpoles in 132 breeding ponds, we examined how the interplay of environmental variables shapes anuran species assemblages in breeding habitats of the dry forest of Western Madagascar. We found that environmental filtering is prevalent and habitat heterogeneity not only increases larval species richness but also alters species composition between these assemblages. Our study highlights the need for conserving heterogenous habitats to maintain local diversity. Furthermore, we recommend including multivariate modelling approaches to conservation efforts to acknowledge differences between specific habitats and beta diversity.

## 1. Introduction

Conservation of pond-breeding amphibians requires monitoring and protection of their terrestrial as well as their aquatic breeding habitats [1,2,3,4]. The degradation and loss of the latter are the main factors leading to declines in amphibian diversity [5]. Even subtle differences in environmental factors can result in major declines in species, if they affect crucial individual needs [6,7]. Alarmingly, two out of three amphibian species are impaired by habitat alterations, such as destruction and fragmentation [5,6]. Nevertheless, limnic habitats are usually not sufficiently considered in conservation plans [4], although their importance for many and in particular rare species has been reported by numerous studies [8,9,10]. Therefore, the IUCN Amphibian Conservation Action Plan highlighted the need for identification, investigation, and protective measures of amphibian habitats [6].

Madagascar has an especially high level of local endemism, with few remnants of undisturbed forest. Due to ongoing deforestation and degradation, many of Madagascar’s species are under threat, particularly in the dry forests of Western Madagascar [4]. Amphibians there fulfill an important functional role in this ecosystem, which makes this area a conservation priority.

Due to their biphasic life cycle as tadpoles and frogs, many amphibians have complex ecological requirements regarding their habitats and the occurrence of these two ecologically very different stages can depend on their surroundings [11,12]. Biotic (e.g., predators, food availability) and abiotic (e.g., pond size, hydroperiod) properties of ponds are among the most important forces affecting species assemblages because they constrain the distribution of anuran species. These properties of ponds can act as filters, preventing or allowing species to colonize and persist in a given habitat [13,14]. Thus, they influence species’ occurrences and, thereby, the assemblage composition of a habitat [7]. The distinct characteristics of amphibians, such as low mobility and permeable skin, make them especially sensitive to environmental filters and vulnerable to changes [15,16,17]. A high variability in environmental filters is equivalent to high heterogeneity between habitats. More heterogenous environments are presumed to support a greater number of species and, consequently, increased species diversity [18,19,20,21]. Identifying environmental characteristics that structure amphibian assemblages and determining suitable habitats are a prerequisite for understanding the ecological underpinnings of amphibians’ lifestyle, and for proposing pertinent conservation plans [1,22,23,24,25,26,27].

**Figure A1:**
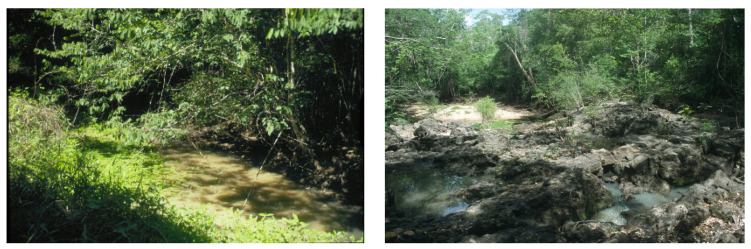
Forest pond (**left**) and riverbed pond (**right**) in Kirindy Forest. Species richness and composition of tadpole assemblages differ between these habitat types.

As greater habitat heterogeneity offers a greater variety of microhabitats and thus niche opportunities that serve the needs of multiple species, we hypothesized that the environmental heterogeneity of breeding ponds a) increases the number of species present within a pond, in the following called species richness, and b) affects which species occur within a pond, here called species composition. Thereby, environmental filters limit the occurrence of some species that cannot cope with the environmental conditions of a certain habitat. They lack the ability to persist in this habitat. Species turnover between ponds that differ in environmental descriptors would indicate for that. Therefore, we tested for differences in environmental variables and species composition between two naturally occurring habitat types within the same forest in Western Madagascar, namely frog breeding ponds within the dry forest and ponds within a riverbed that intersects the forest (riverbed ponds Figure A1 Appendix A). Riverbed ponds usually form after the first heavy rains in November or December and last for up to one and a half months before ongoing rainfall leads to the connection of riverbed ponds before the river starts to flow [28]. Forest ponds usually arise later than ponds in the riverbed, generally in late November or mid-December. Their hydroperiod is very variable (between three days and five months), depending on rainfall. Differences in tadpole assemblage composition between ponds were defined as ß-diversity. We then performed multivariate analyses to evaluate which environmental descriptors of breeding ponds influence species richness and assemblage composition within and between these habitat types.

## 2. Material and Methods

### 2.1. Study Area

Central Menabe, the area between Morondava and the Tsiribinha River in Western Madagascar, has been a “Site de Conservation” since 2006 and covers an area of 1250 km^2^. Located in the center of Menabe, 50 km northeast of Morondava and 20 km inland (44°39′ E, 20°03′ S; 18–40 m above sea level; [29]), the dry, deciduous Kirindy Forest/CNFEREF (Centre National de Formation, d’Etudes et de Recherche en Environnement et Foresterie Morondava) comprises approximately 120 km^2^ [30]. The climate in Kirindy Forest is highly seasonal with a rainy season of three to five months from November/December to February/March, followed by a dry season with virtually no precipitation of seven to nine months. The mean annual precipitation is approximately 800 mm (range 390–1511 mm; data from 1906 to 1993; [29]). The soil has a low capacity to retain water, as it mainly consists of sandy soils [4]. Consequently, most breeding sites for amphibians desiccate completely during the dry season, allowing reproduction only during the rainy season [29]. Ponds in the Kirindy Forest are highly dynamic and heterogenous, with regard to predators and pond size [31], e.g., pond size varies from <5 m^2^ to >10,000 m^2^.

### 2.2. Data Acquisition

#### 2.2.1. The Presence of Anuran Species at Breeding Ponds

A total of 132 potential breeding ponds were sampled (n = 47 forest ponds, n = 85 riverbed ponds) over four consecutive rainy seasons, from 1998 to 2002. These ponds were within a range of 3 km^2^. The use of ponds as breeding sites by anurans was determined by the presence of tadpoles. This method directly determines breeding success and does not rely on the assumption that frog presence or calling activity corresponds to actual breeding at a breeding site.

Tadpoles were sampled by standardized dip-netting [32]: In each pond and in each sampling event, 30 dip net strokes were performed, randomly distributed over the pond. The dip net was triangularly shaped with a base of 400 cm^2^ (30 × 30 × 30 cm; mesh size 1 mm). Each dip net stroke was 1 m long and touched the ground substrate. All tadpoles were identified to the species level in the field camp, using a stereomicroscope and existing literature [33,34]. Most tadpoles were subsequently returned into their natal ponds. Only a few voucher specimens were retained for comparison with a reference collection. 

All ponds were repeatedly sampled every year. Sampling intervals were adapted to detect all species at a certain site irrespective of their breeding mode (i.e., explosive vs. prolonged breeders) and length of larval development, leading to a variable total number of sampling events at each site in one year. To detect tadpoles of explosively breeding species that tend to breed directly after heavy rainfall [31], a sampling event was repeated at each pond approximately one week after each rainfall event that exceeded 30 mm, and after each refilling of ponds after they had dried out. To detect tadpoles of prolonged breeders at breeding ponds with longer hydroperiods, additional sampling was performed in intervals of approximately one month throughout the rainy season. 

Environmental variables and tadpole species composition were consistent between seasons and therefore data from all four years were pooled. Thus, species richness was defined as the total number of species at each breeding site and assemblage composition was defined as the species identities within one pond, irrespective of year and sampling date. 

#### 2.2.2. Environmental Variables of Breeding Ponds

For all ponds in both habitat types (forest and riverbed ponds), a set of nine abiotic and biotic variables was measured at each breeding pond (Table 1), representing measures of important aspects in tadpole ecology such as pond hydroperiod, microhabitat niches and predation. These variables were measured at each pond each year and also repeatedly within one year. All measurements of one variable were pooled and divided by the number of measurements for this variable. These means were used for further calculations. To keep an observer bias at minimum, all variables were measured always by the same person (JG).

### 2.3. Statistical Analysis

Statistical analyses were carried out using the statistical computing software *R* (V4.1.2, R Core Team 2021, Vienna, Austria) and the packages Vegan [35], MASS [36], permute [37] and lattice [38] for bivariate correlations, Wilcoxon-test, Generalized linear model (GLM), Non-metric multidimensional scaling (NMDS) and Mantel tests. Differences in environmental variables between habitat types “forest ponds” and “riverbed ponds” were calculated using non-parametric Wilcoxon signed-rank (W) tests. 

#### 2.3.1. Influence of Environmental Variables on Species Richness

Variable Selection—Spearman correlations were used to identify environmental variables that were highly correlated (r > 0.7, as suggested by [39]), and one variable from a variable pair with high collinearity was excluded from further analysis. This was the case for desiccation risk and maximum depth of water bodies (r = −0.87, *p* < 0.001) and for desiccation risk and the percentage of shallow water (<10 cm) (r = 0.72, *p* < 0.001). Additionally, the shading of ponds, as percentage of the pond shaded at noon, correlated with pond surrounding vegetation (r = 0.57, *p* < 0.001), submerged vegetation (r = −0.3, *p* = 0.03), and leaf litter (r = 0.57, *p* < 0.001). Desiccation risk and the different types of vegetation were considered as the biologically more relevant variables. Therefore, maximum pond depth, the percentage of shallow water (<10 cm), and the ponds’ shading were not included in the statistical models.

Modelling—In order to extract the key habitat factors that predict the choice of breeding waters, a GLM was designed [40]. A model was generated that showed which environmental variables best described a change in species richness. The habitat type and seven independent variables were included in the initial model as predictor variables. A Poisson error distribution with a log link function was used with species richness of tadpoles as the dependent variable [41]. Additionally, interactions between environmental variables and habitat type were included in the initial GLM. Variables and interactions were removed using a stepwise Akaike Information Criterion (stepAIC) approach. Thereby, the initial model was simplified and the best fitted model with the lowest AIC was identified. 

#### 2.3.2. The Influence of Environmental Variables on Assemblage Composition

To analyze for differences in assemblage composition (i.e., species-turnover) between sites and between habitat types (i.e., ß-diversity), data were arranged in matrices including absence (coded as “0”) or presence (coded as “1”) of each species at a respective breeding site. A multivariate analysis of permutational variance (perMANOVA) was performed. One-way permutation analysis was set to 9999 permutations and used to determine significant dissimilarities. Graphical representation was obtained from NMDS [42]. Here, the Bray–Curtis dissimilarity index [43,44] was used as a distance measure and three dimensions were used for illustration. 

To analyze the correlation of environmental variables of breeding sites with their tadpole assemblage composition, data of environmental variables were also arranged in matrices including the respective values (i.e., means of multiple sampling events, see above) of each breeding site. Correlations between the matrices of assemblage composition and environmental variables were identified using a Mantel test [45], and using the Bray–Curtis index [43] as a distance measure for both data matrices. Pearson’s product-moment was used to determine correlations. Significance of Mantel correlations was computed through 9999 permutations.

## 3. Results

### 3.1. Comparison of Forest and Riverbed Ponds

Forest ponds were on average larger and much more variable in size but nevertheless, had a higher desiccation risk and contained relatively more submerged vegetation. The vegetation directly at the edges of the ponds were structurally richer and denser (Table 2). The habitat types did not differ significantly in water turbidity, predator occurrence and leaf litter at the bottom of the ponds. 

### 3.2. Distribution of Tadpole Species 

**Figure A2:**
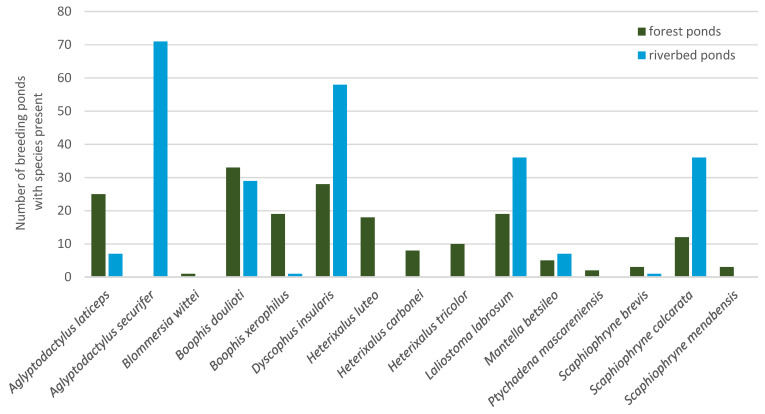
All tadpole species found at 132 ponds within the Kirindy Forest, according to the habitat types: forest ponds (green) and riverbed ponds (blue).

Fifteen species were recorded within 132 ponds, over the duration of four years. We identified tadpoles belonging to the families of Mantellidae (*Aglyptodactylus laticeps*, *A. securifer*, *Blommersia wittei*, *Boophis doulioti*, *Boophis xerophilus*, *Laliostoma labrosum*, *Mantella betsileo*), Microhylidae (*Dyscophus insularis*, *Scaphiophryne brevis*, *S. calcarata*, *S. menabensis*), Hyperoliidae (*Heterixalus carbonei, H. luteostriatus*, *H. tricolor*) and Ptychadenidae (*Ptychadena mascareniensis*) (Figure A2 Appendix A). Species richness in ponds ranged from zero to eleven species (3.27 ± 2.03; mean ± SD). Tadpoles of 14 species in total were recorded in forest ponds (3.96 ± 2.52); nine species in riverbed ponds (2.88 ± 1.58). Tadpoles of six species were only recorded in forest ponds (*Blommersia wittei*, *Heterixalus carbonei*, *H. luteostriatus*, *H. tricolor*, *Ptychadena mascareniensis*, *Scaphiophryne menabensis*) and one species (*A. securifer*) only in riverbed ponds. Eight species occurred in both habitat types. Tadpoles of *D. insularis* were the overall most frequent species, occurring in over 50% of the waterbodies. In forest ponds, tadpoles of *B. doulioti* (n = 33) were the most frequent whereas *A. securifer* was the most frequent species in riverbed ponds (n = 71).

### 3.3. Environmental Variability and Species Richness

When only comparing species richness between habitat types (i.e., not including other environmental variables of the ponds), forest ponds had significantly more species than riverbed ponds (W = 2416.5, *p* = 0.04 (Table 3)). In the model selection process starting with a model including all environmental variables and interactions, six interactions and two variables were removed stepwise from the GLM: Habitat:Surrounding Vegetation (step 1), Habitat:Pond size (step 2), Pond size (step 3), Habitat:Desiccation (step 4), Surrounding Vegetation (step 5), Habitat:Turbidity (step 6) and Habitat:Leaf litter (step 7).

**Figure 1:**
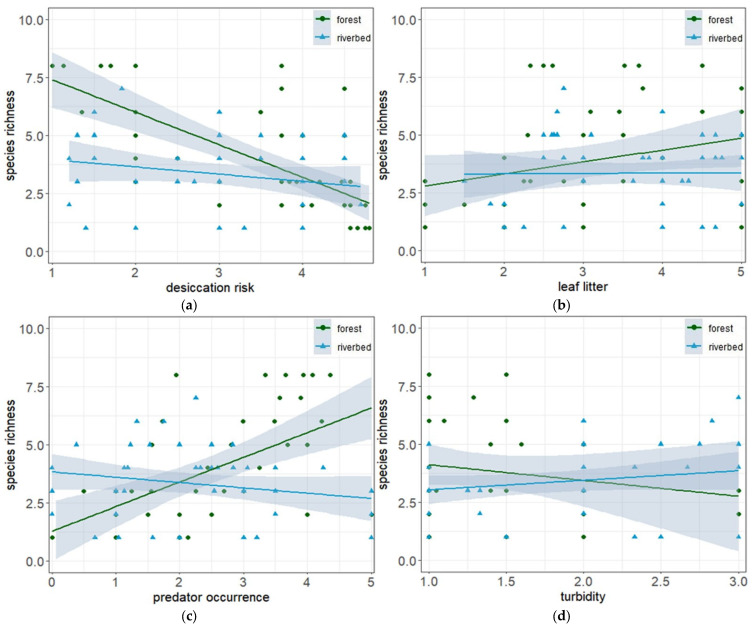
Variation in richness of tadpole species between two habitat types in relation to (**a**) desiccation risk, (**b**) leaf litter, (**c**) predator occurrence and (**d**) pond turbidity. Green dots indicate forest pond plots, blue triangles indicate riverbed ponds. Confidence intervals (95%) are indicated by grey shadows.

Based on the final GLM, four variables and one interaction were significantly associated with tadpole species richness (Table 4). Species richness declined with desiccation risk in both habitat types, although the effect was stronger in forest ponds (Figure 1a). Species richness increased with the amount of leaf litter as substrate, in particular in forest ponds (Figure 1b). Also, the interaction of predator occurrence and habitat type had a significant influence, increasing species richness with predator density in forest ponds but decreasing species richness in riverbed ponds (Figure 1c). Finally, species richness increased with turbidity in riverbed ponds, but decreased in forest ponds (Figure 1d). 

### 3.4. Species Composition in Different Habitat Types

**Figure 2:**
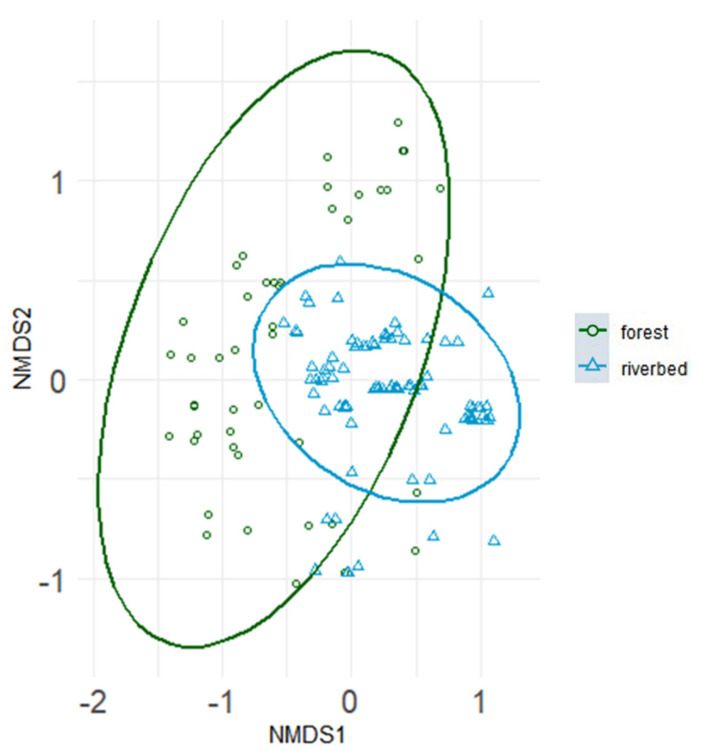
Non-metric multidimensional scaling of tadpole species compositions of forest ponds (green dots) and riverbed ponds (blue triangles); axes 1 and 2 of a three-dimensional analysis. Species communities incorporate absence/presence data. Ellipses indicate 95% confidence intervals fitted into the spatial ordination. R^2^ = 0.55, *p* < 0.001, stress = 0.13.

Species composition differed significantly between forest ponds and riverbed ponds (perMANOVA: R^2^ = 0.55, F = 155.81; *p* < 0.001; Figure 2).

Differences in species composition between breeding ponds were correlated with habitat type, desiccation risk, pond size, submerged vegetation, surrounding vegetation, predator occurrence and leaf litter (Table 4; Mantel test statistics). Only pond turbidity did not show a correlation with species composition.

## 4. Discussion

Frogs have to select breeding ponds to improve survival of their tadpoles. Amphibians of the Kirindy Forest show exceptional responses to their environment [31] and most of them are endemic to this habitat [4,46]. There are currently 15 known amphibian species that use forest and riverbed ponds of the Kirindy Forest as breeding sites [31]. Species such as *A. laticeps* and *S. menabensis* occur exclusively in relatively undisturbed forest patches [4]. Their presence is accompanied by low water permanency and diverse vegetation surrounding the pond, both of which indicate an undisturbed forest. Thus, they function as indicators for an intact environment [4]. They also represent umbrella species (“umbrella effect” [47]) for their habitats because other species including *B. doulioti*, *D. insularis*, *L. labrosum*, *M. betsileo* and *S. calcarata* [33] as well as aquatic species of other taxa, benefit from their protection [4]. However, the availability and nature of breeding ponds depends on environmental conditions and habitat types. Even within the relatively small area of Kirindy Forest, adjacent habitat types contain sufficient environmental heterogeneity to cause species turnover (change in species) between ponds. Thus, adult anurans that select either forest or riverbed ponds will encounter different environmental conditions at their breeding sites. The clear correlations of most environmental variables with assemblage composition indicate that environmental filters we measured were effective in both habitat types, limiting the occurrence of some species and making the site suitable for others. While most species breed in both habitats, a number of species are found almost exclusively either at forest ponds (*Aglyptodactylus laticeps*, *Heterixalus* spp.) or riverbed ponds (*Aglyptodactylus securifer*).

Pond characteristics that were shown to be important variables in determining amphibian assemblage composition included habitat heterogeneity, predation, pond size, surrounding vegetation, submerged vegetation, leaf litter and desiccation risk. Forest ponds with a long hydroperiod, clear water, an abundance of leaf litter and many invertebrates contained more species than others. In riverbed ponds, however, species richness was either un-correlated or only weakly correlated with hydroperiod, leaf litter and predators, but positively correlated with water turbidity.

Previous studies on anuran assemblages showed different effects of environmental heterogeneity at breeding ponds on assemblage composition. Some studies did not detect any environmental influence on assemblage composition [48]. Others not only linked greater habitat heterogeneity to increased community diversity of anurans in aquatic, but also in terrestrial habitats [49,50]. Many studies found that homogenous areas contained fewer species compared to heterogenous sites [51,52,53]. For conservation purposes, it is therefore necessary to develop individual approaches for each area, as no uniform pattern can be assumed. 

Among the variables tested in this study, the size of the breeding pond and the length of the hydroperiod have been previously shown to influence which species select a certain breeding pond [11,20,53,54]. Smaller water bodies usually have shorter hydroperiods and so dry out faster [54]. Therefore, assemblage composition might be related to different lengths of developmental time of the tadpoles. For example, *B. xerophilus,* which has a longer developmental time (approximately 30 days larval duration) and is generally larger as tadpole, is restricted to breeding in larger, more permanent ponds, whilst *B. doulioti*, which shows high developmental plasticity, also uses smaller and more ephemeral ponds for oviposition [55]. Developmental time might depend on the type of reproductive pattern of the species. Most anuran species in the Kirindy Forest are explosive breeders, reproducing only after heavy rainfalls [56]. Their reproductive success depends on fine-tuning the time of oviposition to environmental conditions [57]. Accordingly, species with short larval development such as *A. laticeps* and *Scaphiophryne* spp. (≤10 days; [31,33]) were found predominantly in temporary ponds, which have the advantage of fewer predators compared to permanent ponds. Some prolonged breeders reproduce over longer periods in the rainy season [56]. For example, *H. tricolor* and *H. carbonei*, both prolonged breeders, are specialized to breed in more permanent ponds [58]. Therefore, it is likely that explosive breeders mainly choose ponds with short hydroperiods and a lack of predators, whilst prolonged breeders prefer ponds with longer hydroperiods. This distinction between explosive and prolonged breeders is consistent with several other studies [59,60,61,62]. The dispersal of amphibians as well as the distribution of other species is related to the hydroperiod of available waterbodies [61,63]. Yet, the number of species that were present within a breeding pond was irrespective of the pond size within this study. This suggests that pond size may act as an environmental filter only for some species, namely the ones that require a specific pond size, but not for others that then take the spot of those that cannot cope with this certain pond size.

In addition to beta diversity, species richness can also be related to the risk of desiccation of breeding sites [14,19]. As desiccation risk decreases, more species can exploit a breeding site [60]. Thus, persistent ponds are assumed to have higher species richness than ponds with a shorter hydroperiod [64]. In Kirindy Forest, the choice of breeding sites was considerably more affected by desiccation risk in forest ponds than in riverbed ponds. Riverbed ponds are mainly rock pools with a better capacity to retain water, and thus a lower risk for desiccation. The chance of these drying before the tadpoles have metamorphosed is slim. Indeed, riverbed ponds were usually the first to be used for spawning [28]. 

However, the riverbed ponds present other challenges to tadpoles: strong rainfall raise the water level and eventually the separate ponds merge and the river starts to flow. Invasive cichlid fish (*Oreochromis* sp.) that have spent the dry season in the few permanent ponds of the riverbed are able to move freely throughout the riverbed and increase the risk of predation for tadpoles [28]. Predation can affect communities via (a) direct predation [65,66]; (b) elimination of competitors [67]; and (c) differential predation, depending on prey size [68]. Size-directed predation can favor survivors by reducing competition in an early larval stage [68]. Adult amphibians may then choose breeding sites that show lower competition over sites with low predation which in turn, might lead to a higher species richness in these habitats [68]. Therefore, predation in riverbed ponds reduces the occurrence of all tadpole species equally. Forest ponds, however, stay free from fish predation [28]. In forest ponds, high species richness was observed together with a high occurrence of invertebrate predators (mainly larvae of dragonflies, dytiscid beetles and belostomatid water bugs). This is not an unusual finding. Size-directed predation can favor survivors by limiting individuals in their early metamorphosis, thus reducing competition. Adult amphibians may then choose breeding sites that show lower competition over sites with low predation which in turn, might lead to a higher species richness in these habitats [68].

Vegetation in and around waterbodies and riparian areas can decrease desiccation by maintaining humidity and has been considered an important influence affecting assemblage composition [20,49,51,54]. Vegetation supports sites for vocalization, mating, and oviposition [20] and increases the structural complexity of an environment. Hence, the increased availability of reproductive sites and niches serves the multiple needs of different species of breeding frogs [18,19,69,70]. In forest ponds, vegetation was generally higher compared to riverbed ponds and beta diversity was strongly influenced by these differences. Vegetation structure might influence the choice of breeding sites of anurans in the terrestrial as well the aquatic habitat [71,72]. As a reaction to high predation risks, many species (as frogs) tend to prefer structurally complex pond surroundings for oviposition, but also (as tadpoles) structurally complex sites within ponds [65,73], because most predators hunt visually. In a structurally complex habitat, foraging efficiency of predators is reduced when vegetation functions as shelter for the prey individuals, and thus, the survival of tadpoles significantly increases with denser aquatic vegetation [74,75].

Like vegetation, pond turbidity can also provide visual cover for tadpoles. Water turbidity has a camouflaging effect that decreases the risk of predation, making the pond available for a greater variety of tadpole species [76]. Additionally, turbidity results from an increase in organic sediments, which may serve as a food resource for tadpoles [77]. Indeed, in riverbed ponds, turbidity was accompanied by a high species richness. However, turbidity negatively influenced species richness in forest ponds. This might be an effect of water turbidity also negatively affecting growth rates of tadpoles and survival to metamorphosis [77]. The conflicting effects observed here could demonstrate a trade-off of using the advantages of turbidity as a shelter and/or food resource and its negative effect on growth and/or survival of tadpoles. 

Six out of seven environmental variables that were analyzed here were correlated with species composition, whilst four variables were correlated with species richness. As comparisons with studies in other anuran habitats have shown, relevant variables differ between different habitats and species assemblages. Therefore, different environmental influences must be considered in conservation management to protect unique anuran assemblage in each type of breeding pond.

## 5. Conservation Implications

The heterogeneity between breeding sites was able to support the individual requirements of a vast variety of anuran species. But this also shows that environmental filters were effective in both habitat types and thus limited the occurrence of some species but made the site suitable for those that were capable of coping with the prevailing conditions. Several species of this study, such as *A. laticeps* and *S. menabensis*, are endangered and have a very limited area of occurrence within Western Madagascar. Additionally, they occur exclusively in relatively undisturbed forest patches within their distribution range [4]. These species live on the edge, and their life-history is finely tuned to conditions typical for undisturbed forests. They breed in ponds with occasionally very short hydroperiods of only ten days or less. If such ponds are under the forest canopy and have micro-climatic conditions of an undisturbed forest, the larvae regularly metamorphose just before the pond dries out. In conservation efforts, these species may function as indicators of an intact habitat [4]. They may also represent umbrella species (“umbrella effect” [47]) as several other frog species (such as *B. doulioti*, *D. insularis*, *L. labrosum*, *M. betsileo*, and *S. calcarata*), as well as terrestrial and aquatic species of other taxa, may benefit from their conservation [4].

Due to ongoing deforestation and degradation, many of the species in the Menabe Region are under threat [4]. The development of priority conservation plans is thus a critical step to guide pond conservation strategies, either through regular review of the assignment of important sites, management, and acquisition policy, or implementation of conservation restrictions and other land-use agreements that preserve the variety of breeding sites. This study highlights the need to preserve a variety of ponds with differences in hydroperiod, pond size and vegetation (including surrounding vegetation, submerged vegetation and leaf litter) which provide diverse microhabitats and refugia. These habitat characteristics should be considered when attempting to identify breeding sites that could provide core sites in conservation reserves designed for the conservation of water-breeding amphibians. If breeding sites become unavailable because of a shift in these environmental gradients, not only amphibian diversity but perhaps the whole ecosystem might be harmed.

## Figures and Tables

**Table 1:** List of independent habitat variables recorded at each pond within the Kirindy Forest, including unit, detailed nomenclature and method.

Variable Name	Variable Definition	Unit	Method
Desiccation risk	Desiccation intensity of ponds.	Five categories: 1 (not at risk of desiccation = long hydroperiod) to 5 (extreme risk of desiccation = short hydroperiod)	Estimated by the number of days before desiccation, after a complete filling of the pond and a successive period of no rainfall.
Pond size	Absolute surface area of pond when maximally water filled.	m^2^	Measured using length, width and shape.
Maximum depth	Maximum pond depth when maximally water filled.	cm	Measured at deepest point of water body.
Shallow water	Relative pond area shallower than 10 cm.	%	Visual estimation.
Submerged vegetation	Relative volume of pond filled with submerged water plants.	Six categories: 0 (0%), 1 (1–20%), 2 (21–40%), 3 (41–60%), 4 (61–80%), 5 (81–100%)	Visual estimation.
Surrounding vegetation	Density and complexity of vegetation structure ≤ 2 m from pond edge.	Six categories: 0 (no vegetation) to 5 (high density)	Visual estimation.
Leaf litter	Relative area of pond bottom covered with dead leaves.	Six categories: 0 (0%), 1 (1–20%), 2 (21–40%), 3 (41–60%), 4 (61–80%), 5 (81–100%)	Visual estimation.
Turbidity	Turbidity of the pond water influenced by dissolved and suspensed matters.	Three categories: 1 (clear water), 2 (slightly dull), 3 (very turbid water)	Visual estimation.
Predator occurrence	Visual estimation of invertebrate predators, including: dytiscid beetles > 0.5 cm, larval dytiscid beetles > 1 cm, water bugs (Belostomatidae) > 0.5 cm, water scorpions (Nepidae) > 1 cm, larval dragonflies (Anisoptera) > 0.5 cm, larval damselflies (Zygoptera) > 2 cm.	Six categories: 0 (no predators) to 5 (high density; >5 predators per dip net stroke)	Measured using dip-netting and the box-method [32] and averaged; determined to higher taxonomic levels in the field and subsequently released.

**Table 2:** Environmental differences between forest ponds and riverbed ponds, indicated by means ± standard deviation and results of Wilcoxon-tests. Variables marked with an asterisk are considered to differ significantly between the habitat types.

Variables	Forest Ponds	Riverbed Ponds	Wilcoxon-Test
Desiccation (1 to 5)	3.5 ± 1.2	2.9 ± 0.9	2653.5, *p* = 0.001 *
Pond size (m^2^)	1185.3 ± 3617.6	29.8 ± 62.3	3038.5, *p* < 0.001 *
Submerged vegetation (0 to 5)	1.4 ± 1.2	0.1 ± 0.2	3594.0, *p* < 0.001 *
Surrounding vegetation (0 to 5)	4.2 ± 0.9	2.4 ± 0.8	3630.0, *p* < 0.001 *
Turbidity (1 to 3)	1.4 ± 0.5	1.5 ± 0.7	1997.5, *p* = 1
Predator occurrence (0 to 5)	2.5 ± 1.3	2.1 ± 1.4	1202.5, *p* = 0.25
Leaf litter (0 to 5)	3.2 ± 1.3	3.6 ± 1.0	1595.5, *p* = 0.07

**Table 3:** Initial and final GLM according to a stepAIC approach. Initial model (AIC = 362.3) contained the habitat type, seven environmental variables and interactions of habitat type and each independent variable, respectively. The final model (AIC = 349.9) included habitat type, six environmental variables and two interactions. Variables marked with asterisks are considered significant variables within the model, with * = *p* < 0.05 and ** = *p* < 0.01.

Coefficients	Estimate	Standard Error	z-Value	*p*-Value
Initial generalized linear model (AIC = 362.3)				
Habitat	−0.31	0.11	−0.28	0.78
Desiccation	−0.22	0.12	−1.90	0.06
Leaf litter	0.11	1	1.11	0.27
Predator occurrence	0.17	0.73	2.39	0.02 *
Pond size	−0.38	0.23	−0.16	0.87
Submerged vegetation	0.65	1	0.70	0.51
Surrounding vegetation	−0.33	0.15	−0.22	0.83
Turbidity	1	0.19	0.52	0.60
Habitat:Desiccation	0.6	0.15	0.39	0.7
Habitat:Leaf litter	0.95	0.15	0.65	0.52
Habitat:Predator occurrence	−0.3	0.98	−3.05	<0.001 **
Habitat:Pond size	0.15	0.42	0.35	0.73
Habitat:Subermerged vegetation	0.53	0.42	1.25	0.21
Habitat:Surrounding vegetation	−0.74	0.22	−0.34	0.73
Habitat turbidity	0.2	0.22	0.85	0.73
Final generalized linear model (AIC = 349.9)				
Habitat	0.47	0.3	1.58	0.12
Desiccation	−0.18	0.07	−2.71	<0.01 **
Leaf litter	0.13	0.05	2.44	0.01 *
Turbidity	0.2	0.1	2.09	0.04 *
Predators	0.18	0.07	2.61	0.01 **
Submerged vegetation	0.09	0.08	1.19	0.23
Habitat:Submerged vegetation	0.61	0.37	1.66	0.1
Habitat:Predator occurrence	−0.3	0.09	−3.28	<0.001 **

**Table 4:** Mantel test correlations of environmental variables and tadpole beta diversity of assemblages. Variables marked with an asterisk are considered to significantly correlate with a change in species composition between habitat types.

Variables	Mantel Statistic r	Significance
Habitat	0.43	*p* < 0.001 *
Desiccation	0.13	*p* < 0.001 *
Pond size	0.19	*p* < 0.001 *
Submerged vegetation	0.27	*p* < 0.001 *
Surrounding vegetation	0.28	*p* < 0.001 *
Turbidity	−0.01	0.61
Predator occurrence	0.08	0.02 *
Leaf litter	0.12	*p* < 0.001 *

## Data Availability

Data will be deposited in Dryad at acceptance of the manuscript.
